# *Erratum:* Vol. 66, No. 34

**DOI:** 10.15585/mmwr.mm6644a12

**Published:** 2017-11-10

In “*QuickStats*: Percentage of Children Aged 6–17 Years Who Wear Glasses or Contact Lenses, by Sex and Age Group — National Health Interview Survey, 2016,” on page 917, the caption should have read as follows:

“In 2016, the percentage of children aged 6–17 years who wear eyeglasses or contact lenses was higher among girls (**35.9%**) compared with boys (29.1%). Girls aged 6–9 years (**20.8%**) and 14-17 years (**51.4%**) were more likely than boys of the same age group (**15.4%** and **38.1%**, respectively) to wear eyeglasses or contact lenses. There was no statistically significant difference by sex for children aged 10-13 years (**35.4%** among girls, **33.7%** among boys). Among both girls and boys, children aged 14–17 years were most likely to wear eyeglasses or contact lenses and children aged 6–9 years were least likely to wear eyeglasses or contact lenses.”

